# Changes in forced vital capacity over ≤ 13 years among patients with late-onset Pompe disease treated with alglucosidase alfa: new modeling of real-world data from the Pompe Registry

**DOI:** 10.1007/s00415-024-12489-9

**Published:** 2024-06-19

**Authors:** Kenneth I. Berger, Yin-Hsiu Chien, Alberto Dubrovsky, Priya S. Kishnani, Juan C. Llerena, Edward Neilan, Mark Roberts, Bun Sheng, Julie L. Batista, Magali Periquet, Kathryn M. Wilson, Ans T. van der Ploeg

**Affiliations:** 1 Division of Pulmonary, Critical Care and Sleep Medicine, NYU Grossman School of Medicine, and the André Cournand Pulmonary Physiology Laboratory, Bellevue Hospital, New York, NY USA; 2 Department of Medical Genetics, National Taiwan University Hospital, Taipei, Taiwan; 3 Department of Neurology, Neuromuscular Disease Unit, Institute of Neuroscience, Favaloro Foundation, Buenos Aires, Argentina; 4 Division of Medical Genetics, Department of Pediatrics, Duke University Medical Center, Durham, NC USA; 5 Centro de Genética Médica, Instituto Fernandes Figueira/FIOCRUZ, Rio de Janeiro, Brazil; 6 National Organization for Rare Disorders (NORD®), Quincy, MA USA; 7 Salford Royal NHS Foundation Trust, Salford, UK; 8 Department of Medicine & Geriatrics, Princess Margaret Hospital, Lai Chi Kok, Hong Kong; 9 Sanofi, Cambridge, MA USA; 10 UCB—Rare Diseases Organization, Brussels, Belgium; 11 Navitas Data Sciences, Pottstown, PA USA; 12 Center for Lysosomal and Metabolic Diseases, Erasmus MC, University Medical Center, Rotterdam, The Netherlands

**Keywords:** Forced vital capacity, Late-onset Pompe disease, Alglucosidase alfa, Observational study, Real-world evidence, Pompe Registry

## Abstract

**Background:**

Chronic respiratory insufficiency from progressive muscle weakness causes morbidity and mortality in late-onset Pompe disease (LOPD). Previous Pompe Registry (NCT00231400) analyses for ≤ 5 years’ alglucosidase alfa treatment showed a single linear time trend of stable forced vital capacity (FVC) % predicted.

**Methods:**

To assess longer term Pompe Registry data, piecewise linear mixed model regression analyses estimated FVC% predicted trajectories in invasive-ventilator-free patients with LOPD aged ≥ 5 years. We estimated annual FVC change 0–6 months, > 6 months–5 years, and > 5–13 years from treatment initiation, adjusting for baseline age, sex, and non-invasive ventilation.

**Findings:**

Among 485 patients (4612 FVC measurements; 8.3 years median follow-up), median ages at symptom onset, diagnosis, and alglucosidase alfa initiation were 34.3, 41.1, and 44.9 years, respectively. FVC% increased during the first 6 months’ treatment (slope 1.83%/year; 95% confidence interval: 0.66, 3.01; *P* = 0.0023), then modestly declined −0.54%/year (−0.79, −0.30; *P* < 0.0001) during > 6 months–5 years, and −1.00%/year (−1.36, −0.63; *P* < 0.0001) during > 5–13 years. The latter two periods’ slopes were not significantly different from each other (*P*_difference_ = 0.0654) and were less steep than published natural history slopes (−1% to −4.6%/year). Estimated individual slopes were ≥ 0%/year in 96.1%, 30.3%, and 13.2% of patients during the 0–6 month, > 6 month–5 year, and > 5–13 year periods, respectively.

**Conclusion:**

These real-world data indicate an alglucosidase alfa benefit on FVC trajectory that persists at least 13 years compared with published natural history data. Nevertheless, unmet need remains since most individuals demonstrate lung function decline 5 years after initiating treatment. Whether altered FVC trajectory impacts respiratory failure incidence remains undetermined.

**Trial registration:**

This study was registered (NCT00231400) on ClinicalTrials.gov on September 30, 2005, retrospectively registered.

**Supplementary Information:**

The online version contains supplementary material available at 10.1007/s00415-024-12489-9.

## Introduction

The clinical course of respiratory muscle function has been evaluated in several studies of subjects with late-onset Pompe disease (LOPD) during enzyme replacement therapy with alglucosidase alfa [1–3]. Clinical trials reported a slight increase in forced vital capacity (FVC) over the initial 6 months of alglucosidase alfa treatment [2, 4]. However, the subsequent evolution of respiratory function remains unclear. While some studies and systematic reviews reported stabilization of lung function for up to 5 years during alglucosidase alfa treatment [4, 5], other observational reports [1, 6, 7] indicated a gradual decline in vital capacity, particularly at timepoints beyond 5 years.

The present study analyzes Pompe Registry upright FVC data with more detailed methods in a pediatric and adult cohort over a longer duration to answer three fundamental questions: (1) Do real-world data corroborate an increase in FVC in the first 6 months of alglucosidase alfa treatment? (2) What is the course of FVC over longer time frames from > 6 months to 13 years on treatment? (3) Do longitudinal patterns of FVC differ based on age at diagnosis, baseline FVC, or time from diagnosis to first treatment?

## Methods

### Study design and oversight

The Pompe Registry (NCT00231400) was initiated in 2004 and is a long-term, multi-national, observational program in patients with Pompe disease that is sponsored and managed by Sanofi (Cambridge, MA, USA). Physician investigators may enroll patients into the Registry irrespective of patients’ age, clinical presentations, treatment, or clinical trial participation status provided there is a confirmed Pompe diagnosis. Registry sites are encouraged to enter all patient data; however, these data do vary among patients both in the types of data collected and in their frequency of collection according to patient needs, local practices, resources, and available skills.

Each participating center obtained written informed consent from patients or, for minors, their guardians, to submit their data to the Pompe Registry and to use and disclose this information anonymously in aggregate analyses. Local sites’ Institutional Review Boards or Independent Ethics Committees approved the Pompe Registry protocol, informed consent form, and local authorization documents for data submissions.

### Study population

The study included data downloaded from the Pompe Registry as of September 2021 for patients with LOPD aged ≥ 5 years at treatment initiation with alglucosidase alfa. The ≥ 5-year-old inclusion criterion was chosen to be consistent with a previous analysis and because children younger than 5 years may have difficulty performing an accurate FVC maneuver [5]. LOPD was defined as symptom onset at ≤ 12 months of age without cardiac enlargement/cardiomyopathy or symptom onset at > 12 months of age [8].

For patients’ data to be included in the analysis, patients had to have a baseline upright FVC value measured between 6 months before and 1 month after treatment initiation, along with ≥ 2 additional FVC records in the period from 1 month to 13 years after first treatment (giving a total of ≥ 3 FVC values per patient). Only upright FVC was included in the analysis, as there were fewer supine FVC data available, and supine measurements can be prone to bias if positional dyspnea interferes with patients’ ability to perform the test optimally. At least 6 months were required between the first and last FVC measurement. Measurements beyond 13 years from first treatment were not included as only < 5% of patients had a record beyond this time limit.

Exclusion criteria, which paralleled those of a prior Pompe Registry analysis [5], were invasive ventilation at alglucosidase alfa initiation or an implausible annual change in a patient’s FVC, as defined by a change in slope, estimated by linear regression, across all their measurements from baseline through follow-up on alglucosidase alfa of > +10% or < −10%/year. Patients whose first treatment was not alglucosidase alfa were excluded. Patients who switched from alglucosidase alfa to a different treatment were included in the analysis until the date of the switch.

### Study measures

Demographic and clinical information including FVC measurements over time was collected in case report forms. Date of symptom onset was derived from the earliest of (1) diagnosis date reported for ophthalmic, respiratory, gastrointestinal/hepatic, renal, or musculoskeletal symptoms related to Pompe disease; (2) date of respiratory support use, date of ambulatory symptoms (ambulatory with difficulty, ambulation lost, wheelchair use), or date of cardiac enlargement/cardiomyopathy from echocardiogram or chest X-ray (onset at > 12 months of age for this study cohort’s definition of individuals with LOPD); or (3) directly reported age of onset of Pompe disease symptoms. Diagnosis date was derived from the earliest of confirmatory enzyme assay date, genotype assay date, or physician-reported diagnosis date (directly reported by the Registry site).

### Statistical analyses

#### Demographics and baseline clinical characteristics

Descriptive analyses were generated for demographic and clinical variables overall and according to categories of age at baseline (≥ 5 to < 18 years or ≥ 18 years) and FVC % predicted at baseline (< 55%, 55 to 80%, or > 80%). The baseline FVC categories chosen were based on prior Pompe disease studies [﻿^2^, ^5^, ^9^] and published guidelines for spirometry testing [10, 11]. Medians, 25th and 75th percentiles, and ranges are presented for continuous variables.

#### Longitudinal change in FVC during alglucosidase alfa treatment

Upright FVC was analyzed using % predicted values. Change in FVC over time was assessed using linear mixed effects models to account for repeated measures within patients over time. To assess how change in FVC over time might vary based on time from treatment initiation, we used piecewise linear regression to estimate the annual change in FVC over three periods: 0 to 6 months, > 6 months to 5 years, and > 5 to 13 years from treatment initiation. The periods were chosen a priori based on results from clinical trials that suggest an initial increase in FVC after treatment initiation in alglucosidase alfa-naïve patients [2] and previous observational studies (including from the Pompe Registry) that found a period of stability in the first 5 years of treatment [1, 5]. We fitted separate slopes for the > 6 months to 5 years and > 5 to 13 years periods to assess whether the change in FVC in the later period was different from that seen in the first 5 years after treatment initiation.

The final models included fixed effects for: time from treatment initiation (providing an estimate of the slope for the initial 0 to 6 months of treatment), two linear spline terms to allow the slopes to vary for the > 6 months to 5 years and > 5 to 13 years periods, baseline age, sex, and use of non-invasive respiratory support at baseline, and random effects for: the intercept (FVC at baseline), time, and the two linear spline terms for time. The slopes from the three time-related variables give the estimated annual change in FVC in absolute percentage points/year (%/year). An unstructured covariance matrix was used to accommodate the varying number of measurements and timing of measurements across patients, and models were estimated using restricted maximum likelihood (REML). Fixed effects for age, sex, and baseline non-invasive ventilation were included, as all three were significantly associated with baseline FVC and their inclusion improved model fit as assessed by the Akaike Information Criterion.

In addition to estimating FVC slopes in the full study population, we assessed whether slopes in the three periods varied according to age at treatment initiation (≥ 5 to < 18 years or ≥ 18 years), baseline FVC (< 55%, 55 to 80%, or > 80%), or time from diagnosis to first treatment (below or at/above the median time for adult patients, < 1.5 or ≥ 1.5 years, respectively) using interaction terms between the three time-related variables and indicator variables for the subgroups. The median time from diagnosis to first treatment was selected as a cut-off because, in a prior shorter-term Pompe Registry analysis [5], a shorter time from diagnosis to first treatment was associated with a higher FVC.

For the models by baseline FVC category, the first period was extended to 1 year instead of 6 months after treatment initiation due to limited data within each baseline FVC subgroup. Thus, results for this analysis present estimated changes in FVC for the periods of 0 to 1 year, > 1 to 5 years, and > 5 to 13 years from treatment initiation. To test for overall significant differences in FVC slopes between subgroups, we used likelihood ratio tests comparing models with and without interaction terms between the patient subgroup and the time variables. Likelihood ratio tests were based on the maximum likelihood estimation of the linear mixed models, as REML methods cannot be used to test fixed effects.

We assessed the robustness of the results with sensitivity analyses excluding patients with very low FVC at baseline (< 30%), excluding patients diagnosed before 2006 (when alglucosidase alfa became widely available—prior to 2006, alglucosidase alfa was available only in clinical trials or for compassionate use), and excluding follow-up measurements from > 10 to 13 years after treatment initiation (as less than one-quarter of individuals, 106/485 (21.9%), had an assessment beyond 10 years).

To assess the variation in patients’ FVC responses in each period, we calculated individual predicted slopes for each patient using both the fixed and random effects estimates from the main model. The results are presented as box plots.

All statistical analyses were performed using SAS (version 9.4; SAS Institute Inc., Cary, NC, USA), and *P* values < 0.05 were considered statistically significant.

## Results

### Patient disposition

**Fig. 1:**
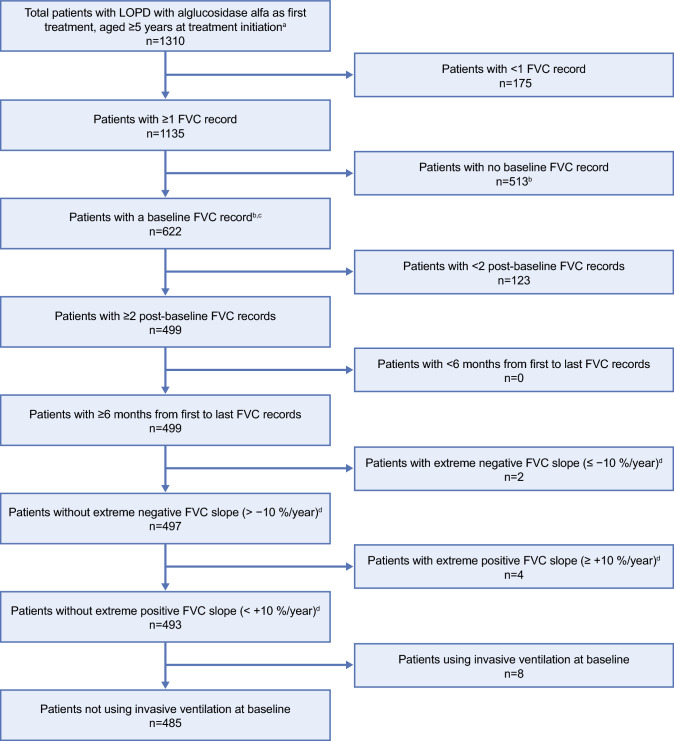
Patient disposition for the total cohort. ^a^Patients must also have non-missing date of diagnosis. ^b^Baseline measurement is the closest to alglucosidase alfa start date, within a window of 6 months before to 1 month after that date. ^c^Only one baseline record per patient is included in the analysis. ^d^Annual change across all measurements. *FVC* forced vital capacity, *LOPD* late-onset Pompe disease

Patient disposition is shown in Fig. 1. Of the 1310 patients identified, 622 had multiple FVC measurements including presence of a baseline value. After consideration of all inclusion/exclusion criteria, 485/622 (78%) patients remained, comprising the final study population. These eligible patients had in total 4612 FVC records over the 13 years of follow-up. Two hundred (41.2%) patients had in total two hundred twenty-eight FVC records in the post-baseline period from 1 to 6 months after first treatment, four hundred seventy-eight (98.6%) had two thousand three hundred ninety-eight FVC records in the period from > 6 months to 5 years after first treatment, and three hundred thirty-nine (69.9%) had one thousand five hundred one FVC records in the period > 5 to 13 years after first treatment.

### Demographics and baseline clinical characteristics

Baseline demographics, Pompe disease diagnosis, and treatment data for the overall study population and according to age at first treatment and baseline FVC category are shown in Table 1. Median age at LOPD symptom onset was 34.3 years, at LOPD diagnosis was 41.1 years, and at first treatment with alglucosidase alfa was 44.9 years. Approximately 10.3% of patients were ≥ 5 to < 18 years old at first treatment. Patients with a higher baseline FVC were diagnosed and treated at earlier ages and had a shorter time from diagnosis to first treatment. The proportions of patients diagnosed before 2006 were similar for the baseline FVC < 55% and 55 to 80% groups. More of those with a baseline FVC > 80% compared with the other baseline FVC groups had been diagnosed after 2006, in the era of available treatment. Most patients (94.7% at their most recent record) received around the label dose of alglucosidase alfa, 20 mg/kg every other week.

**Table 1:** Baseline demographics, Pompe disease diagnosis, and treatment data for the analysis population

Parameter	All patients	Age at baseline		FVC category at baseline		
		< 18 years	≥ 18 years	< 55%	55% to 80%	> 80%
Total patients, N	485	50	435	155	188	142
Male, n (%)	246 (50.7)	30 (60.0)	216 (49.7)	106 (68.4)	92 (48.9)	48 (33.8)
Region, n (%)						
Europe, Middle East, Africa	330 (68.0)	29 (58.0)	301 (69.2)	96 (61.9)	131 (69.7)	103 (72.5)
Japan and Asia–Pacific	20 (4.1)	10 (20.0)	10 (2.3)	6 (3.9)	9 (4.8)	5 (3.5)
Latin America	7 (1.4)	0	7 (1.6)	2 (1.3)	2 (1.1)	3 (2.1)
North America	128 (26.4)	11 (22.0)	117 (26.9)	51 (32.9)	46 (24.5)	31 (21.8)
Age at symptom onset: patients with data, n	467	47	420	152	183	132
Median (25th, 75th percentiles), years	34.3 (17.5, 45.1)	9.1 (4.6, 13.8)	36.2 (22.8, 46.1)	33.1 (16.2, 44.9)	36.5 (17.5, 48.5)	31.7 (18.3, 41.6)
Range (min, max), years	0.0, 75.7	0.0, 21.2	0.0, 75.7	0.0, 67.8	0.0, 75.7	0.0, 70.1
Age at LOPD diagnosis: patients with data, n	485	50	435	155	188	142
Median (25th, 75th percentiles), years	41.1 (29.7, 53.0)	9.6 (3.1, 14.2)	42.9 (34.6, 54.5)	42.7 (29.3, 53.7)	44.1 (32.2, 56.5)	37.7 (25.9, 45.9)
Range (min, max), years	0.3, 82.5	0.3, 17.0	0.7, 82.5	0.7, 76.1	1.7, 82.5	0.3, 75.2
Diagnosis age < 18 years, n (%)	69 (14.2)	50 (100)	19 (4.4)	22 (14.2)	19 (10.1)	28 (19.7)
Diagnosis age ≥ 18 years, n (%)	416 (85.8)	0	416 (95.6)	133 (85.8)	169 (89.9)	114 (80.3)
Diagnosed via newborn screening	1 (0.2)	1 (2.0)	0	1 (0.6)	0	0
Time period of diagnosis: patients with data, n	485	50	435	155	188	142
Before 2006	207 (42.7)	20 (40.0)	187 (43.0)	76 (49.0)	89 (47.3)	42 (29.6)
2006 to 2010	142 (29.3)	17 (34.0)	125 (28.7)	42 (27.1)	50 (26.6)	50 (35.2)
2011 to 2015	115 (23.7)	12 (24.0)	103 (23.7)	31 (20.0)	42 (22.3)	42 (29.6)
2016 to 2021	21 (4.3)	1 (2.0)	20 (4.6)	6 (3.9)	7 (3.7)	8 (5.6)
Age at first treatment: patients with data, n	485	50	435	155	188	142
Median (25th, 75th percentiles), years	44.9 (34.4, 57.4)	13.1 (9.9, 15.5)	47.0 (39.0, 58.6)	48.3 (36.6, 58.6)	47.1 (35.5, 60.4)	40.7 (29.1, 50.0)
Range (min, max), years	5.8, 83.0	5.8, 17.6	18.2, 83.0	8.5, 76.5	5.8, 83.0	6.0, 75.5
First treated age < 18 years, n (%)	50 (10.3)	50 (100)	0	11 (7.1)	14 (7.4)	25 (17.6)
First treated age ≥ 18 years, n (%)	435 (89.7)	0	435 (100)	144 (92.9)	174 (92.6)	117 (82.4)
Time from symptom onset to first treatment: patients with data, n	467	47	420	152	183	132
Median (25th, 75th percentiles), years	9.7 (3.6, 17.0)	2.9 (0.0, 6.6)	10.3 (4.3, 17.9)	12.0 (5.6, 19.7)	10.3 (4.6, 17.9)	5.7 (1.6, 12.1)
Range (min, max), years	0.0, 60.1	0.0, 12.8	0.0, 60.1	0.0, 58.6	0.0, 60.1	0.0, 51.6
Time from diagnosis to first treatment: patients with data, n	485	50	435	155	188	142
Median (25th, 75th percentiles), years	1.4 (0.4, 7.1)	0.7 (0.2, 7.7)	1.5 (0.4, 7.1)	2.0 (0.5, 9.7)	1.7 (0.4, 7.4)	0.9 (0.4, 3.2)
Range (min, max), years	0.0, 30.9	0.0, 15.0	0.0, 30.9	0.0, 30.9	0.0, 24.9	0.0, 21.0
Time from first treatment to most recent follow-up^a^: patients with data, n	485	50	435	155	188	142
Median (25th, 75th percentiles), years	8.3 (5.5, 11.4)	8.8 (6.3, 11.9)	8.3 (5.5, 11.3)	8.2 (6.1, 11.3)	8.6 (5.0, 11.5)	8.1 (5.3, 11.3)
Range (min, max), years	0.9, 16.3	2.7, 16.3	0.9, 15.1	1.5, 15.0	1.0, 15.2	0.9, 16.3
Most recent alglucosidase alfa dose: patients with data, n^b^	475	49	426	152	186	137
Very low dose, n (%)	4 (0.8)	0	4 (0.9)	1 (0.7)	1 (0.5)	2 (1.5)
Label dose, n (%)	450 (94.7)	44 (89.8)	406 (95.3)	144 (94.7)	174 (93.5)	132 (96.4)
40 mg/kg qow, n (%)	12 (2.5)	3 (6.1)	9 (2.1)	5 (3.3)	6 (3.2)	1 (0.7)
20 mg/kg qw or higher, n	0	0	0	0	0	0

Baseline is the date of treatment initiation

*FVC* forced vital capacity, *LOPD* late-onset Pompe disease

^a^Most recent follow-up is the date of the most recent Registry assessment or date of death

^b^Dose categories: ‘Very low dose’: > 0 to < 14 mg/kg qow or qw, ‘Label dose’: around the label dose of 20 mg/kg qow, range of 14 to 27 mg/kg qow, ’40 mg/kg qow’: > 27 to 52 mg/kg qow, ’20 mg/kg qw or higher’: 14 to 52 mg/kg qw. Doses > 52 mg/kg (of any frequency) were excluded

Table 2 shows ambulatory, respiratory, and mortality data for the overall study population and according to age at first treatment and baseline FVC category. Approximately one-quarter of patients used non-invasive ventilation at baseline (27.9% of the patients with a recorded baseline ventilatory status). Of the patients with a recorded ambulatory status at baseline, 54.3% were ambulatory with difficulty and 5.6% were non-ambulatory. Thirty-four (7.0%) patients were reported deceased during the follow-up period, with a median age at death of 60.3 years.

**Table 2:** Ambulatory, respiratory, and mortality data for the analysis population

Parameter	All patients	Age at baseline		FVC category at baseline		
		< 18 years	≥ 18 years	< 55%	55% to 80%	> 80%
Non-invasive ventilation at baseline: patients with data, n	477	49	428	151	186	140
Yes, n (%)	133 (27.9)	3 (6.1)	130 (30.4)	95 (62.9)	33 (17.7)	5 (3.6)
Ambulatory status at baseline: patients with data, n	479	50	429	152	187	140
Ambulatory, n (%)	192 (40.1)	34 (68.0)	158 (36.8)	50 (32.9)	63 (33.7)	79 (56.4)
Ambulatory with difficulty, n (%)	260 (54.3)	14 (28.0)	246 (57.3)	82 (53.9)	119 (63.6)	59 (42.1)
Non-ambulatory, n (%)	27 (5.6)	2 (4.0)	25 (5.8)	20 (13.2)	5 (2.7)	2 (1.4)
Wheelchair use at baseline: patients with data, n	479	50	429	152	187	140
Yes, n (%)	31 (6.5)	4 (8.0)	27 (6.3)	18 (11.8)	8 (4.3)	5 (3.6)
6MWT distance at baseline: patients with data, n (%)^a^	286 (59.0)	28 (56.0)	258 (59.3)	84 (54.2)	113 (60.1)	89 (62.7)
Median (25th, 75th percentiles), m	380 (285, 476)	520 (449, 610)	369 (274, 456)	304 (203, 411)	375 (288, 468)	457 (360, 538)
FVC at baseline: patients with data, n^b^	485	50	435	155	188	142
Median (25th, 75th percentiles), %	67.1 (49.5, 85.0)	80.4 (65.0, 91.1)	65.5 (49.0, 83.1)	41.0 (32.0, 49.0)	68.6 (61.8, 75.0)	93.5 (87.7, 100.6)
Number of FVC records/patient, median (25th, 75th percentiles)	8 (6, 13)	10 (7, 14)	8 (5, 12)	9 (6, 13)	8 (5, 12)	7 (5, 12)
Time from baseline to most recent FVC measurement: patients with data	485	50	435	155	188	142
Median (25th, 75th percentiles), years	6.9 (4.6, 9.8)	7.9 (5.4, 11.0)	6.8 (4.5, 9.8)	6.7 (4.8, 9.0)	7.6 (4.5, 10.2)	7.0 (4.6, 9.8)
Deceased during follow-up, n (%)	34 (7.0)	0	34 (7.8)	21 (13.5)	11 (5.9)	2 (1.4)
Age at death, years, median (25th, 75th percentiles)	60.3 (52.5, 69.3)	–	60.3 (52.5, 69.3)	65.5 (53.7, 69.3)	54.2 (50.1, 66.6)	56.4 (41.4, 71.3)

Baseline is the date of treatment initiation

*6MWT* 6-min walk test, *FVC* forced vital capacity (assessed as % predicted), *qow* every other week, *qw* every week

^a^The baseline 6MWT assessment was the one closest to the date of treatment initiation and within a window of 1 year before to 1 year after that

^b^The baseline FVC assessment was the one closest to the date of treatment initiation and within a window of 6 months before to 1 month after that date

Overall, five (1.0%) patients included in the study reported invasive ventilation during follow-up; only two of these patients had FVC measurements from time points after the initiation of invasive ventilation. The post-invasive ventilation data for the two patients are included in the current analysis.

Patients aged ≥ 5 to < 18 years at baseline had less severe disease than those aged ≥ 18 years, with lower proportions of non-invasive ventilation use and ambulatory impairment and without reported deaths during follow-up. Similarly, patients in the highest baseline FVC groups (> 80% or 55 to 80% predicted) had less severe disease at baseline with lower proportions of non-invasive ventilation and ambulatory impairment coupled with a decreased likelihood of death during follow-up as compared with individuals with FVC < 55% predicted (Table 2).

### FVC measurements

In the full study population, median baseline FVC was 67.1% (Table 2). Median baseline FVC was higher among patients aged ≥ 5 to < 18 years at baseline (80.4% predicted) compared with those aged ≥ 18 years (65.5% predicted). The median number of available FVC measurements per patient was 8, with a maximum of 28 measurements per patient. The median number of records per patient was slightly higher (10 records) for patients aged ≥ 5 to < 18 years vs ≥ 18 years at baseline (8 records) and for patients with baseline FVC values < 55% predicted (9 records) than those with 55 to 80% predicted (8 records) or > 80% predicted (7 records). The median follow-up time from first to last FVC measurement was 6.9 years. Follow-up time was slightly longer for patients aged ≥ 5 to < 18 years than those ≥ 18 years at baseline (medians 7.9 and 6.8 years, respectively). Follow-up time was similar across baseline FVC categories.

#### Longitudinal change in FVC during alglucosidase alfa treatment

**Fig. 2:**
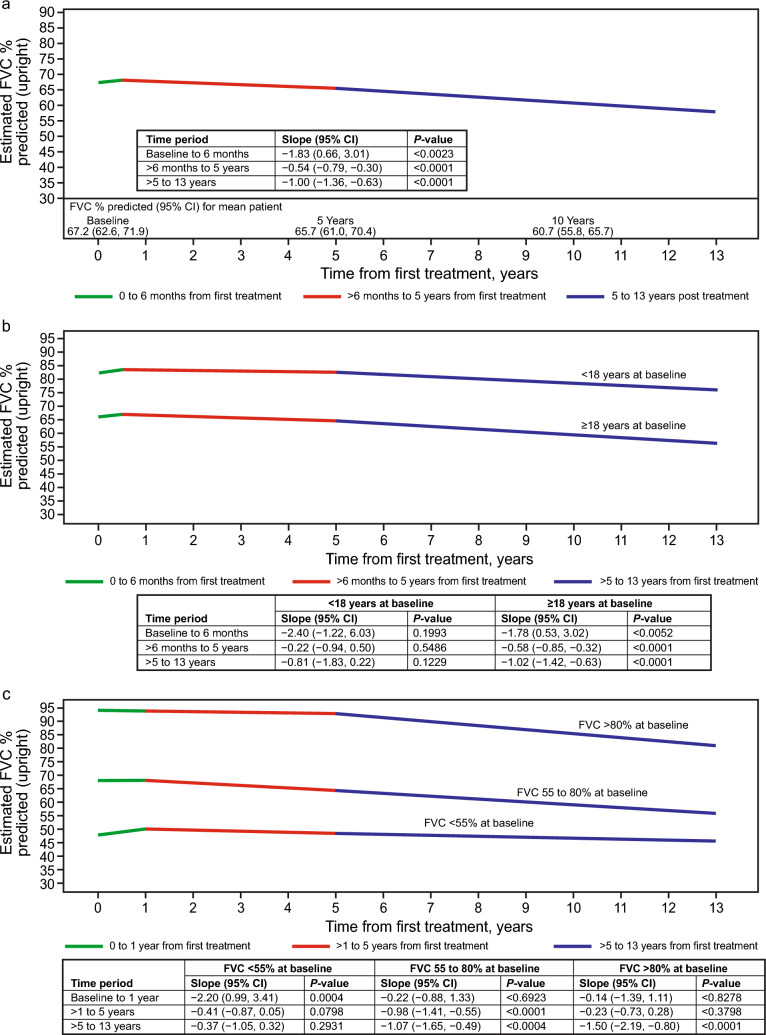
Estimated upright FVC (as % predicted) over time from treatment initiation, based on a linear mixed model in alglucosidase alfa-treated patients with LOPD. The model is adjusted for baseline age, sex, and use of non-invasive respiratory support at baseline. Slopes for each line segment represent the estimated annual change in FVC%/year. The number of patients and number of FVC assessments available in each period are provided in the “Patient disposition” section of the Results. **a** Overall study cohort: the figure shows the estimated FVC over time for a patient with baseline FVC equal to the mean baseline value as predicted by the linear mixed model for the study population (67.2%). While the estimated baseline FVC (y-axis intercept) will vary based on covariate values, the depicted slopes (annual change in FVC) are constant across covariate values. **b** By age category at first treatment. Estimated FVC over time for patients with baseline FVC equal to the mean predicted baseline value for their age group (75.5% for < 18 years, 66.2% for ≥ 18 years at baseline). Likelihood ratio test for significant difference in slopes between groups: *P* = 0.6228. **c** By baseline FVC category: estimated FVC over time for patients with baseline FVC equal to the mean predicted baseline value for their FVC group (41.9% for < 55%, 68.1% for 55 to 80%, and 94.0% for > 80% at baseline). Likelihood ratio test for significant difference in slopes between groups: *P* = 0.0003. *CI* confidence interval, *FVC* forced vital capacity (as % predicted), *LOPD* late-onset Pompe disease

For the overall study population, data for estimated change in FVC over time after alglucosidase alfa initiation are summarized in Table 3 and illustrated in Fig. 2. FVC increased during the first 6 months of treatment, with an estimated slope of 1.83%/year (95% confidence interval [CI]: 0.66, 3.01, *P* = 0.0023). Thereafter, FVC declined in the period > 6 months to 5 years (estimated slope: −0.54%/year [95% CI—0.79, −0.30], *P* < 0.0001) and in the period > 5 to 13 years (estimated slope: −1.00%/year [95% CI—1.36, −0.63], *P* < 0.0001). The estimated decline for > 5 to 13 years appeared steeper than that for > 6 months to 5 years; however, the difference between these slopes was not statistically significant (*P* = 0.0654). The sensitivity analyses yielded similar results to the estimates for the overall study population (supplemental information: Table S1).

**Table 3:** Linear mixed model estimates^a^ of FVC % predicted slopes over time since alglucosidase alfa treatment initiation for the full LOPD study population, and stratified by age at first treatment, baseline FVC, and time from diagnosis to treatment

Model	Number of patients (number of FVC records)	FVC slope estimate, %/year	95% CI	SE	*P* value
All patients	485 (4612)				
Baseline to 6 months		1.83	0.66, 3.01	0.597	**0.0023**
> 6 months to 5 years		−0.54	−0.79, −0.30	0.125	**< 0.0001**
> 5 to 13 years		−1.00	−1.36, −0.63	0.187	**< 0.0001**
Difference: > 5 to 13 years vs > 6 months to 5 years		−0.452			0.0654
Patients separated by age at first treatment					
< 18 years	50 (564)				
Baseline to 6 months		2.40	−1.22, 6.03	1.843	0.1933
> 6 months to 5 years		−0.22	−0.94, 0.50	0.365	0.5486
> 5 to 13 years		−0.81	−1.83, 0.22	0.520	0.1229
Difference: > 5 to 13 years vs > 6 months to 5 years		−0.587			0.3914
≥ 18 years	435 (4048)				
Baseline to 6 months		1.78	0.53, 3.02	0.632	**0.0052**
> 6 months to 5 years		−0.58	−0.85, −0.32	0.133	** < 0.0001**
> 5 to 13 years		−1.02	−1.42, −0.63	0.201	** < 0.0001**
Difference: > 5 to 13 years vs > 6 months to 5 years		−0.437			0.0967
Patients separated by baseline FVC category					
FVC < 55%	155 (1549)				
Baseline to 1 year		2.20	0.99, 3.41	0.616	**0.0004**
> 1 to 5 years		−0.41	−0.87, 0.05	0.234	0.0798
> 5 to 13 years		−0.37	−1.05, 0.32	0.347	0.2931
Difference: > 5 to 13 years vs > 1 to 5 years		0.045			0.9227
FVC 55 to 80%	188 (1771)				
Baseline to 1 year		0.22	−0.88, 1.33	0.563	0.6923
> 1 to 5 years		−0.98	−1.41, −0.55	0.220	** < 0.0001**
> 5 to 13 years		−1.07	−1.65, −0.49	0.295	**0.0004**
Difference: > 5 to 13 years vs > 1 to 5 years		−0.093			0.8182
FVC > 80%	142 (1292)				
Baseline to 1 year		−0.14	−1.39, 1.11	0.637	0.8278
> 1 to 5 years		−0.23	−0.73, 0.28	0.257	0.3798
> 5 to 13 years		−1.50	−2.19, −0.80	0.352	** < 0.0001**
Difference: > 5 to 13 years vs > 1 to 5 years		−1.274			**0.0085**
Patients separated by time from diagnosis to first treatment					
< 1.5 years	248 (2134)				
Baseline to 6 months		1.74	0.10, 3.39	0.837	**0.0381**
> 6 months to 5 years		−0.43	−0.78, −0.07	0.181	**0.0187**
> 5 to 13 years		−1.11	−1.70, −0.51	0.301	**0.0003**
Difference: > 5 to 13 years vs > 6 months to 5 years		−0.680			0.0750
≥ 1.5 years	237 (2478)				
Baseline to 6 months		1.90	0.22, 3.57	0.852	**0.0266**
> 6 months to 5 years		−0.65	−0.98, −0.31	0.172	**0.0002**
> 5 to 13 years		−0.92	−1.39, −0.44	0.240	**0.0002**
Difference: > 5 to 13 years vs > 6 months to 5 years		−0.272			0.4004

*CI* confidence interval, *FVC* forced vital capacity (as % predicted), *LOPD* late-onset Pompe disease, *SE* standard error

^a^All model results are adjusted for baseline age (continuous), sex, and use of non-invasive respiratory support at baseline

Bold type indicates *P* values < 0.05

Models allowing different slopes according to age group at baseline (≥ 5 to < 18 years and ≥ 18 years) showed that none of the slopes for the pediatric age group were statistically different from zero, possibly due to the small sample size of 50 patients (Table 3, Fig. 2). The results for the ≥ 18 years baseline age group were very similar to the overall results, with a significant increase in FVC from 0 to 6 months and statistically significant declines after 6 months, but without statistical difference between the 6 months to 5 years and > 5 to 13 years periods. Likelihood ratio test for significant difference in slopes between groups was *P* = 0.6228 (Fig. 2b).

Differences in FVC trajectories were evident for patients with different baseline FVC values. The first period was extended from 6 months to 1 year for model stability. Patients with baseline FVC < 55% had a significant increase in the period of 0 to 1 years, followed by small and non-significant declines in FVC from > 1 to 5 years and > 5 to 13 years (Table 3, Fig. 2). Patients in the 55 to 80% baseline FVC group had stable FVC in the 0 to 1 year period but had statistically significant declines of approximately 1%/year in both the > 1 to 5 years and > 5 to 13 years periods. Patients with baseline FVC > 80% were stable in the 0 to 1 year and > 1 to 5 years periods and then had a statistically significant decline of 1.5%/year from > 5 to 13 years; for this group alone, the slopes in the > 1 to 5 years and > 5 to 13 years periods were significantly different from each other (*P* = 0.0085).

The overall *P* value for the interaction between baseline FVC category and FVC slopes was 0.0003. Comparing specific slopes within a given period, the 0 to 1 year slope in the < 55% baseline FVC group was significantly different from the slope in that period for patients in the 55 to 80% group (*P* = 0.0184), with a larger initial increase in the < 55% group. The > 1 to 5 years slope for patients with FVC > 80% at baseline was significantly different from the slope in the 55 to 80% group for the same period (*P* = 0.0266), with a slower (and non-statistically significant) decline in the > 80% group.

The longitudinal FVC slopes did not differ in individuals that initiated therapy within 1.5 years of diagnosis versus those that initiated therapy later (differences between groups < 0.22% predicted/year during each follow-up period).

**Fig. 3:**
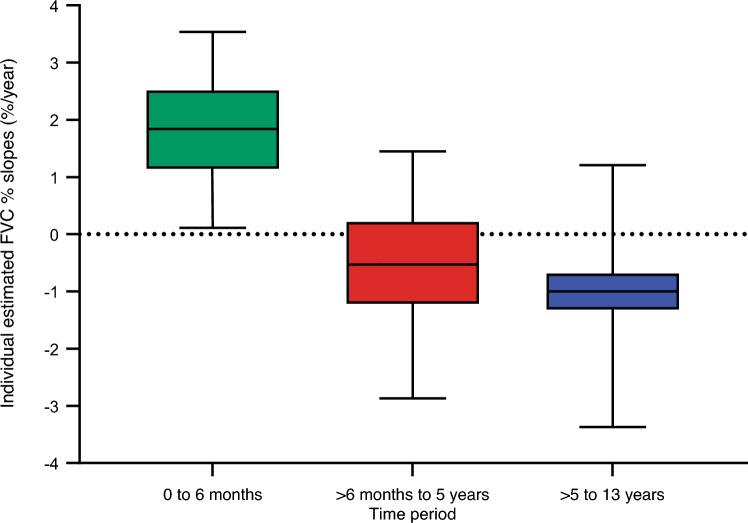
Distribution of individual estimated FVC % predicted slopes from a linear mixed model of FVC % over time from first treatment. Estimates for individual FVC slopes (change in FVC in %/year) are calculated for each patient for each of the three periods using fixed and random effect estimates from the main linear mixed model based on all patients in the study population. Median (5th, 95th percentile) estimated slope in each period: 1.84 (0.11, 3.54) %/year for 0 to 6 months; −0.53 (−2.87, 1.45) %/year for > 6 months to 5 years; and −1.00 (−3.37, 1.21) %/year for > 5 to 13 years. Percent of patients with estimated slope ≥ 0%/year for each period: 96.1% for 0 to 6 months; 30.3% for > 6 months to 5 years; and 13.2% for > 5 to 13 years. Boxes represent the 25th percentile, median, and 75th percentile, and whiskers represent the 5th and 95.^th^ percentiles. *FVC* forced vital capacity (as % predicted)

Estimated individual FVC % slope distributions are shown in Fig. 3 for each of the three periods. For the 0 to 6 months period, individual slope estimates had a median of 1.84%/year. Nearly all patients (96.1%) demonstrated positive or stable slopes (≥ 0%/year) during the 0 to 6 month period (Fig. 3), confirming results from clinical trials and systematic reviews of enzyme replacement therapy with alglucosidase alfa [2–4]. For the > 6 months to 5 years period, individual slope estimates shifted into the negative range for most of the population, with a median of −0.53%/year and 69.7% patients had an estimated slope < 0%/year. FVC slopes for > 5 to 13 years were slightly more negative than those for > 6 months to 5 years, with a median of −1.00%/year, and 86.8% patients had an estimated slope < 0%/year.

## Discussion

This long-term prospective study examined real-world evidence obtained by the Pompe Registry to define the longitudinal course of respiratory function, as assessed by FVC % predicted, in 485 patients with LOPD over 13 years following treatment initiation with alglucosidase alfa. The current analysis builds on a previous study of FVC [5] during the first 5 years of alglucosidase alfa treatment in pediatric and adult patients. The key new findings of our current study relate to the characterization of respiratory function trajectories during different periods after treatment initiation. We confirmed, using real-world data, that a significant increase in FVC occurs during the first 6 months of treatment, in accordance with findings reported during clinical trials and a systematic review [2–4]. In addition, we demonstrated a modest decline in respiratory function from > 6 months to 13 years that matches prior clinical observations ﻿[1, 4, 6, 7, 12]. The declines in FVC in the periods > 6 months to 5 years and > 5 to 13 years were not significantly different from each other, suggesting that respiratory function does not decline significantly more rapidly with longer duration of treatment. Notably, the rate of FVC decline from > 6 months to 5 years and from 5 to 13 years (−0.54%/year and −1.00%/year, respectively) was generally less steep than prior observations in untreated patients (−1.0 to −4.6%/year) [13–16]. Lastly, analysis of individual trajectories confirmed that the majority of subjects experience an increase in respiratory function with the initiation of alglucosidase alfa treatment with smaller numbers of patients showing increases beyond the first 6 months.

The present study further extends prior observations by assessing whether the evolution of lung function during alglucosidase alfa treatment varies by specific patient characteristics at baseline. Patients with baseline respiratory function in the normal range (baseline FVC > 80% predicted) had a long 5-year period of stability following treatment initiation. In these individuals, an increase in lung function immediately after treatment initiation could not be expected given their baseline normal values, but notably, conservation of their existing respiratory function was observed for several years thereafter. In contrast, patients with the most severe respiratory impairment at baseline (FVC < 55% predicted) had room for improvement and demonstrated the largest increase in lung function during the initial 6 months of treatment. These benefits in the patients severely impaired at baseline persisted over the ensuing 13 years, as evidenced by the subsequent slope of decline being less steep than FVC decline in the natural history of untreated Pompe disease [13–16].

Lastly, there was a suggestion that patients < 18 years old at baseline (treatment initiation) had a larger initial increase and slower long-term decline than patients aged ≥ 18 years at baseline; however, our power to detect significant differences between age groups was limited due to the small number of patients aged < 18 years at baseline. Taken together, these data demonstrate a benefit of alglucosidase alfa treatment on respiratory function that is evident in all FVC and age cohorts, with differing manifestations for each group. These observations are reported for group mean data within each subgroup discussed above; individual responses may vary.

Numerous prior studies have evaluated the evolution of respiratory function in patients with LOPD during treatment with alglucosidase alfa [2–4, 7, 9]. These studies differ with respect to baseline patient characteristics, cohort size, and data analytic techniques. Nevertheless, several studies included techniques to assess for varying lung function slope over time and have demonstrated an initial increase in FVC after treatment initiation in treatment-naïve patients with similar magnitude to the present data [2–4, 7, 9]. Specifically, our observation of a slope of 1.83%/year during the first 6 months of alglucosidase alfa treatment was nearly identical to the previous studies’ estimates ranging from 1.8 to 2.8% predicted over time frames ranging from 2 months to 1 year after treatment initiation. In the present piecewise linear modeling analysis, our use of a pre-defined baseline value for FVC with proximity to the start of treatment maximizes the confidence in our estimates of the initial change in lung function in the 0 to 6-month timeframe.

In contrast, slopes for longer-term decline in respiratory function were highly variable between studies. The Late-Onset Pompe Observational Study [14] followed 58 adults for 1 year, finding a mean upright FVC change of −4.6%/year. Whereas, additional studies with durations ranging from 14 months [16] to 16 years [13] reported mean changes in FVC ranging from −1.0 to −1.6%/year.

The present study provides information that may help clarify the etiology for the discrepancies in the reported long-term evolution of lung function. We leveraged data collected from the largest longitudinal and multi-national cohort of patients with Pompe disease. Robust analytic techniques were used to define time courses over discrete time frames from initiation of alglucosidase alfa treatment coupled with subgroup analyses, as defined by differing baseline characteristics. Our subgroup analyses confirm that while the evolution of lung function during alglucosidase alfa therapy is generally better than the untreated natural history, variability is noted between patients, which may explain the differences noted in prior studies. Although the estimated evolution of lung function over long time frames varies across studies, all available data agree that the average decline in FVC during alglucosidase alfa treatment is more gradual than the natural history of the disease. Since the need for ventilatory support is tightly linked to the level of respiratory function [17], the relatively more favorable natural history of disease following initiation of alglucosidase alfa treatment would be expected to delay the need for either nocturnal or daytime respiratory support.

The strengths of this study include its larger real-world sample compared with any preceding study, follow-up extending beyond 5 years to up to 13 years (other studies of long duration have fewer patients at > 5 years), and separate slope segments estimated for treatment durations beyond 5 years to determine if longer treatment experience affects the rate of change. Limitations may include the characteristics of Registry real-world data, including variation in FVC testing techniques across Pompe Registry sites; varying numbers of data points, irregular testing intervals, and/or incomplete data capture due to the practice-based collection of Registry data, and the absence of a direct untreated comparator group. Finally, we used the mixed modeling approach to estimate individual FVC slopes for each patient to understand the range of individual trajectories, but the data do not provide insight into the factors underlying variability across patients. These results do not take into account a patient’s genotype, antibody status, or other possible effect modifiers. The impacts of lung comorbidities and smoking history could not be evaluated as data were not collected in the Registry. Nevertheless, the present data represent the most comprehensive evaluation of long-term lung function during disease-modifying treatment for LOPD, which can inform evolving observations during newly approved and additional investigational therapies that aim to build on and sustain the initial improvement in FVC observed with alglucosidase alfa treatment [18–22].

## Conclusion

This analysis of long-term real-world FVC data demonstrates a benefit of alglucosidase alfa on respiratory function, which is durable and clinically meaningful in comparison with published data on the untreated natural course of LOPD. At a group level, a beneficial response to alglucosidase alfa persists for at least 13 years after treatment initiation. Modeling FVC over the three periods confirmed that respiratory function increases over the first 6 months of treatment in real-world use of alglucosidase alfa, as has been observed in clinical trials. Importantly, while respiratory function appeared to decline modestly with longer duration of treatment, the longitudinal course was much improved as compared with the published natural history of LOPD. These findings indicate a clear and persistent clinical benefit of alglucosidase alfa treatment, as alteration of the lung function trajectory over time would be expected to delay the need for either nocturnal or daytime respiratory support. Nevertheless, an unmet need persists since most individuals demonstrate a decline in lung function 5 years after initiating treatment, which may be addressed by future therapies.

## Supplementary Information

Below is the link to the electronic supplementary material.

Supplementary file1 (DOCX 59 KB)

## Data Availability

The data that support the findings of this study are available to Pompe Registry participants in aggregate format and can be requested through a Data Analyses Request form. The data are not publicly available due to privacy or ethical restrictions. For additional information, please contact rarediseaseregistries@sanofi.com.
