# Correction: Changes in forced vital capacity over ≤ 13 years among patients with late-onset Pompe disease treated with alglucosidase alfa: new modeling of real-world data from the Pompe Registry

**DOI:** 10.1007/s00415-025-13147-4

**Published:** 2025-07-31

**Authors:** Kenneth I. Berger, Yin-Hsiu Chien, Alberto Dubrovsky, Priya S. Kishnani, Juan C. Llerena, Edward Neilan, Mark Roberts, Bun Sheng, Julie L. Batista, Magali Periquet, Kathryn M. Wilson, Ans T. van der Ploeg

**Affiliations:** 1 Division of Pulmonary, Critical Care and Sleep Medicine, NYU Grossman School of Medicine, and the André Cournand Pulmonary Physiology Laboratory, Bellevue Hospital, New York, NY USA; 2 Department of Medical Genetics, National Taiwan University Hospital, Taipei, Taiwan; 3 Department of Neurology, Neuromuscular Disease Unit, Institute of Neuroscience, Favaloro Foundation, Buenos Aires, Argentina; 4 Division of Medical Genetics, Department of Pediatrics, Duke University Medical Center, Durham, NC USA; 5 Centro de Genética Médica, Instituto Fernandes Figueira/FIOCRUZ, Rio de Janeiro, Brazil; 6 National Organization for Rare Disorders (NORD®), Quincy, MA USA; 7 Salford Royal NHS Foundation Trust, Salford, UK; 8 Department of Medicine & Geriatrics, Princess Margaret Hospital, Lai Chi Kok, Hong Kong; 9 Sanofi, Cambridge, MA USA; 10 UCB—Rare Diseases Organization, Brussels, Belgium; 11 Navitas Data Sciences, Pottstown, PA USA; 12 Center for Lysosomal and Metabolic Diseases, Erasmus MC, University Medical Center, Rotterdam, The Netherlands

**Correction: Journal of Neurology (2024) 271:5433–5446** 10.1007/s00415-024-12489-9

In the original version of this article, in Fig. 2 (a-c) in which some of the slope values were incorrectly presented as negative.

Figure 2, which previously appeared as

**Fig. 2:**
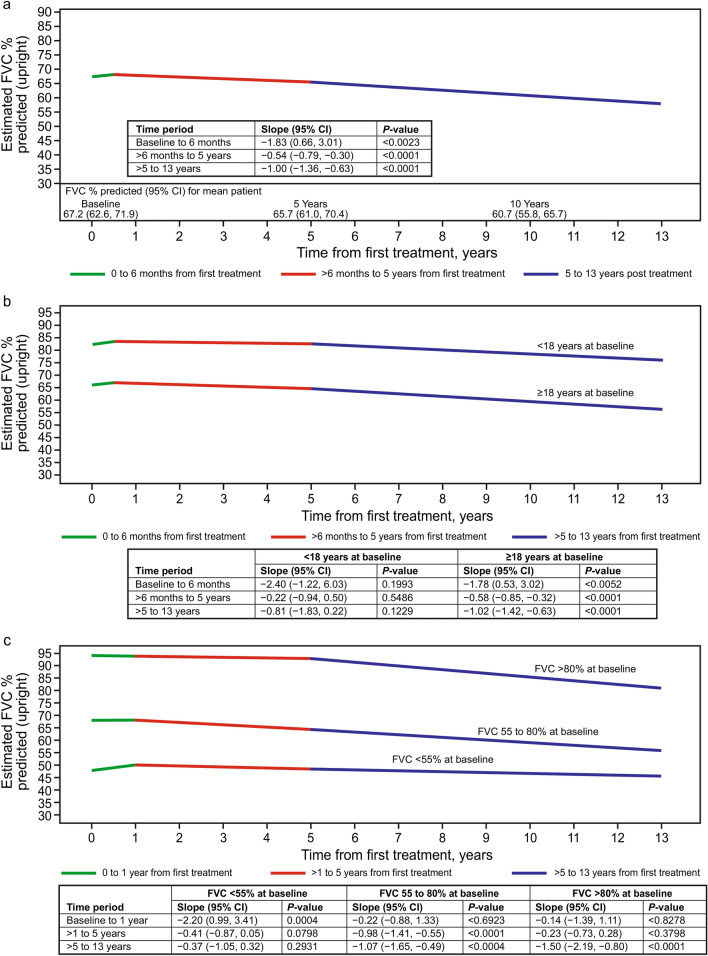
Estimated upright FVC (as % predicted) over time from treatment initiation, based on a linear mixed model in alglucosidase alfatreated patients with LOPD. The model is adjusted for baseline age, sex, and use of non invasive respiratory support at baseline. Slopes for each line segment represent the estimated annual change in FVC%/year. The number of patients and number of FVC assessments available in each period are provided in the “Patient disposition” section of the Results. **a** Overall study cohort: the figure shows the estimated FVC over time for a patient with baseline FVC equal to the mean baseline value as predicted by the linear mixed model for the study population (67.2%). While the estimated baseline FVC (y-axis intercept) will vary based on covariate values, the depicted slopes (annual change in FVC) are constant across covariate values. **b** By age category at first treatment. Estimated FVC over time for patients with baseline FVC equal to the mean predicted baseline value for their age group (75.5% for < 18 years, 66.2% for ≥ 18 years at baseline). Likelihood ratio test for significant difference in slopes between groups: *P* = 0.6228. **c** By baseline FVC category: estimated FVC over time for patients with baseline FVC equal to the mean predicted baseline value for their FVC group (41.9% for < 55%, 68.1% for 55 to 80%, and 94.0% for > 80% at baseline). Likelihood ratio test for significant difference in slopes between groups: *P* = 0.0003. *CI* confidence interval, *FVC* forced vital capacity (as % predicted), *LOPD* late-onset Pompe disease

and figure 2 should have appeared as shown below (correction of previously shown negative slopes in embedded tables)

**Fig. 2:**
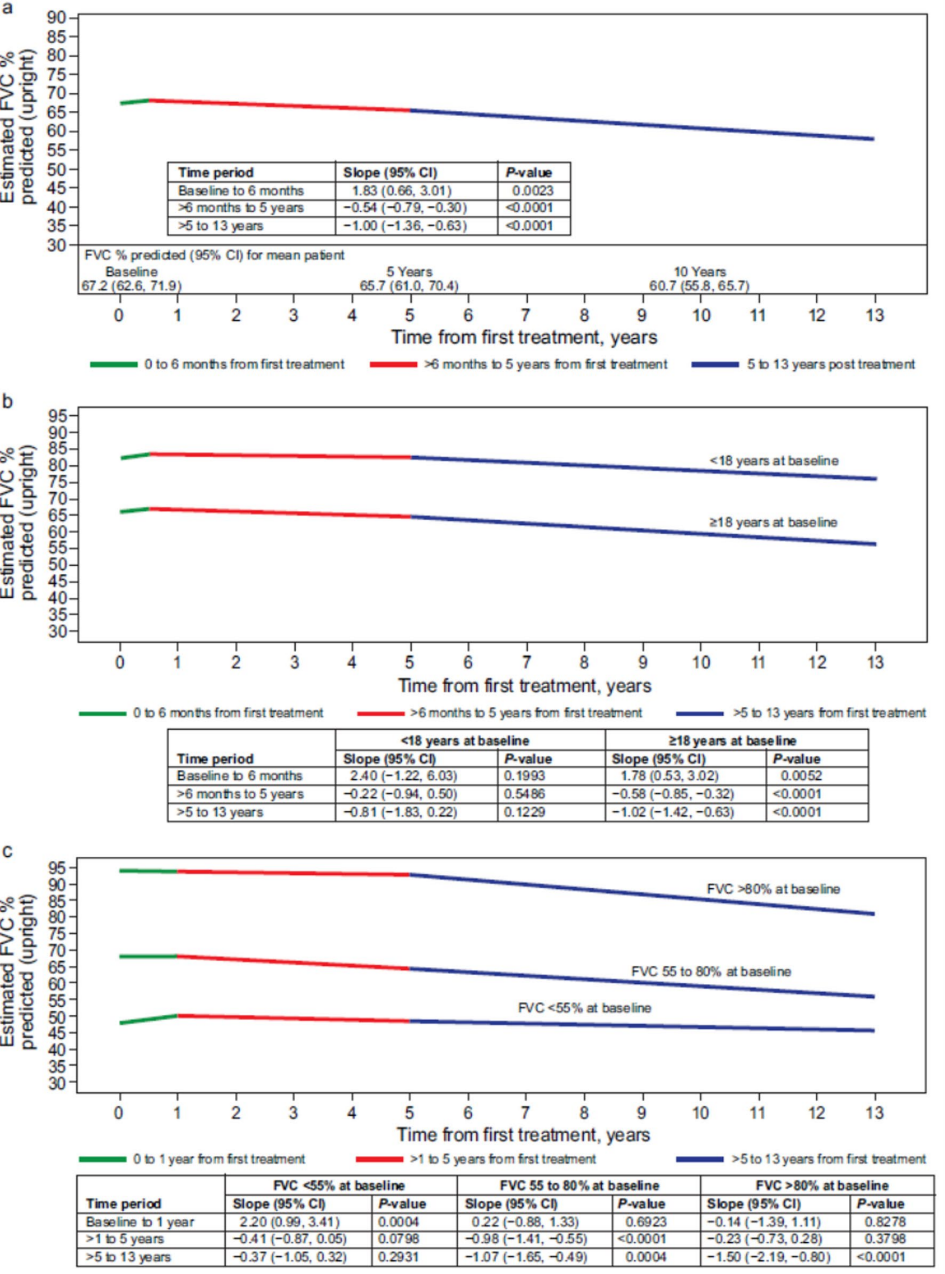
Estimated upright FVC (as % predicted) over time from treatment initiation, based on a linear mixed model in alglucosidase alfatreated patients with LOPD. The model is adjusted for baseline age, sex, and use of non-invasive respiratory support at baseline. Slopes for each line segment represent the estimated annual change in FVC%/year. The number of patients and number of FVC assessments available in each period are provided in the “Patient disposition” section of the Results. **a** Overall study cohort: the figure shows the estimated FVC over time for a patient with baseline FVC equal to the mean baseline value as predicted by the linear mixed model for the study population (67.2%). While the estimated baseline FVC (y-axis intercept) will vary based on covariate values, the depicted slopes (annual change in FVC) are constant across covariate values. **b** By age category at first treatment. Estimated FVC over time for patients with baseline FVC equal to the mean predicted baseline value for their age group (75.5% for < 18 years, 66.2% for ≥ 18 years at baseline). Likelihood ratio test for significant difference in slopes between groups: *P* = 0.6228. **c** By baseline FVC category: estimated FVC over time for patients with baseline FVC equal to the mean predicted baseline value for their FVC group (41.9% for < 55%, 68.1% for 55 to 80%, and 94.0% for > 80% at baseline). Likelihood ratio test for significant difference in slopes between groups: *P* = 0.0003. *CI* confidence interval, *FVC* forced vital capacity (as % predicted), *LOPD* late-onset Pompe disease

