# Supplementary material for: Changes in forced vital capacity over ≤ 13 years among patients with late-onset Pompe disease treated with alglucosidase alfa: new modeling of real-world data from the Pompe Registry
Source: J Neurol. 2024 Jun 19;271(8):5433–46. doi: 10.1007/s00415-024-12489-9 (PMC11319528; doi:10.1007/s00415-024-12489-9)
Supplement: Supplementary file 1 — Supplementary file1 (DOCX 59 KB) [file 415_2024_12489_MOESM1_ESM.docx]

**Changes in forced vital capacity over ≤13 years among patients with late-onset Pompe disease treated with alglucosidase alfa: new modelling of real-world data from the Pompe Registry**

**Supplemental Information**

**Table S1** Estimated FVC % predicted slopes over time since first treatment from linear mixed models:^a^ Sensitivity analyses

| **Model** | **Number of patients**  **(number of FVC records)** | **FVC slope estimate, %/year** | **95% CI** | **SE** | ***P*-value** |
| --- | --- | --- | --- | --- | --- |
| **All patients** | 485 (4612) |  |  |  |  |
| Baseline to 6 months |  | 1.83 | 0.66, 3.01 | 0.597 | **0.0023** |
| >6 months to 5 years |  | −0.54 | −0.79, −0.30 | 0.125 | **<0.0001** |
| >5 to 13 years |  | −1.00 | −1.36, −0.63 | 0.187 | **<0.0001** |
| Difference: >5 to 13 years vs >6 months to 5 years |  |  |  |  | 0.0654 |
| **Excluding baseline FVC <30%** | 456 (4347) |  |  |  |  |
| Baseline to 6 months |  | 1.71 | 0.49, 2.92 | 0.618 | **0.0060** |
| >6 months to 5 years |  | −0.60 | −0.85, −0.35 | 0.127 | **<0.0001** |
| >5 to 13 years |  | −1.06 | −1.44, −0.69 | 0.190 | **<0.0001** |
| Difference: >5 to 13 years vs >6 months to 5 years |  |  |  |  | 0.0716 |
| **Excluding patients diagnosed before 2006** | 278 (2342) |  |  |  |  |
| Baseline to 6 months |  | 1.67 | 0.10, 3.23 | 0.794 | **0.0366** |
| >6 months to 5 years |  | −0.55 | −0.90, −0.21 | 0.175 | **0.0018** |
| >5 to 13 years |  | −1.09 | −1.71, −0.47 | 0.310 | **0.0007** |
| Difference: >5 to 13 years vs >6 months to 5 years |  |  |  |  | 0.1796 |
| **Excluding follow-up beyond 10 years** | 485 (4360) |  |  |  |  |
| Baseline to 6 months |  | 1.87 | 0.70, 3.04 | 0.595 | **0.0018** |
| >6 months to 5 years |  | −0.56 | −0.81, −0.31 | 0.125 | **<0.0001** |
| >5 to 13 years |  | −0.94 | −1.39, −0.50 | 0.226 | **<0.0001** |
| Difference: >5 to 13 years vs >6 months to 5 years |  |  |  |  | 0.1780 |

^a^All model results are adjusted for baseline age (continuous), sex, and use of non-invasive respiratory support at baseline

*CI* confidence interval, *FVC* forced vital capacity (as % predicted), *SE* standard error
